# Clinical relevance and therapeutic potential of angiopoietin-like protein 4 in hepatocellular carcinoma

**DOI:** 10.1186/1476-4598-13-196

**Published:** 2014-08-22

**Authors:** Kevin Tak-Pan Ng, Aimin Xu, Qiao Cheng, Dong Yong Guo, Zophia Xue-Hui Lim, Chris Kin-Wai Sun, Jeffrey Hon-Sing Fung, Ronnie Tung-Ping Poon, Sheung Tat Fan, Chung Mau Lo, Kwan Man

**Affiliations:** Department of Surgery and Centre for Cancer Research, LKS Faculty of Medicine, The University of Hong Kong, Room L9-55, Li Ka Shing Faculty of Medicine Building, 21 Sassoon Road, Pokfulam, Hong Kong, SAR, China; Department of Medicine, LKS Faculty of Medicine, The University of Hong Kong, Room L9-55, Li Ka Shing Faculty of Medicine Building, 21 Sassoon Road, Pokfulam, Hong Kong, SAR, China

**Keywords:** Hepatocellular carcinoma, Angiopoietin-like 4, Angiogenesis, Metastasis, Therapeutic

## Abstract

**Background:**

Development of novel adjuvant therapy to eradicate tumor angiogenesis and metastasis is a pressing need for patients with advanced hepatocellular carcinoma (HCC). We aimed to investigate the clinical relevance and therapeutic potential of angiopoietin-like 4 (ANGPTL4) in HCC.

**Methods:**

*ANGPTL4* mRNA levels in tumor and non-tumor liver tissues of HCC patients were analyzed to investigate its clinical relevance. The mechanisms of deregulation of ANGPTL4 in HCC were studied by copy number variation (CNV) and CpG methylation analyses. The orthotopic liver tumor nude mice model was applied using a human metastatic cell line. ANGPTL4-overexpressing adenovirus (Ad-ANGPTL4) was injected *via* portal vein to investigate its anti-tumorigenic and anti-metastatic potentials.

**Results:**

HCC tissues expressed significantly lower levels of *ANGPTL4* mRNA than non-tumor tissues. The copy number of *ANGPTL4* gene in tumor tissues was significantly lower than in non-tumor tissues of HCC patients. Higher frequency of methylation of CpG sites of *ANGPTL4* promoter was detected in tumor tissues compared to non-tumor tissues. Downregulation of *ANGPTL4* mRNA in HCC was significantly associated with advanced tumor stage, presence of venous infiltration, poor differentiation, higher AFP level, appearance of tumor recurrence, and poor postoperative overall and disease-free survivals of HCC patients. Treatment with Ad-ANGPTL4 significantly inhibited the *in vivo* tumor growth, invasiveness and metastasis by promoting tumoral apoptosis, inhibiting tumoral angiogenesis and motility, and suppressing tumor-favorable microenvironment. Moreover, administration of recombinant ANGPTL4 protein suppressed the motility of HCC cells and altered the secretion profile of cytokines from macrophages.

**Conclusion:**

ANGPTL4 is a diagnostic and prognostic biomarker for HCC patients and a potential therapeutic agent to suppress HCC growth, angiogenesis and metastasis.

## Background

Hepatocellular carcinoma (HCC) is one of the most malignant tumors and the fourth leading cause of cancer-related death worldwide [1]. Surgical operations including liver resection and liver transplantation are effectively curative treatments for patients with early stage of HCC, but patients with advanced HCC cannot be beneficial from it. In addition to clinical shortcoming of chemoresistance of HCC causing ineffective implementation of systemic chemotherapy and targeted therapy, patients with advanced HCC have been often suffering from poor prognosis [2–5]. Therefore, searching for novel adjuvant therapies targeting HCC progression and metastasis is a pressing need.

Angiopoietin-like 4 (ANGPTL4) protein, a secreted protein, is one of the members of angiopoietin (ANG)-relating family which shares very high similarity to the structure of ANG family. ANGPTL4 protein contains a highly hydrophobic signal peptide, an N-terminal coiled-coil domain and a C-terminal fibrinogen-like domain [6]. ANGPTL4 is expressed highly in numerous organs including adipose tissue, liver, heart and small intestine [7–9]. Moreover, ANGPTL4 can be stimulated by inflammatory and hypoxic conditions [6, 7, 10]. ANGPTL4 exerts multifunctional roles such as glucose and lipid metabolisms, inflammation, differentiation, angiogenesis, and tumorigenesis [6, 7]. The roles of ANGPTL4 in human cancers are controversial. Overexpression of ANGPTL4 can promote tumorigenesis, tumor invasion, angiogenesis, anoikis resistance and metastasis [11–15]. On the other hand, ANGPTL4 is an anti-metastatic protein on tumor cells through inhibition of vascular permeability, motility and invasiveness [16]. The clinical implications and functional roles of ANGPTL4 in HCC so far are not well defined. One study has demonstrated that high level of serum ANGPTL4 protein in HCC patients is significantly associated with liver cirrhosis, higher histological grade and intrahepatic metastasis [17]. However, a recent study demonstrated that the expression levels of ANGPTL4 protein in tumor tissues are significantly lower than in non-tumor tissues of HCC patients [18]. Our previous study has demonstrated that ANGPTL4 plays important roles in regulating glucose and lipid metabolisms of the liver in mice [19]. In this study, we aimed to investigate the clinical relevance of ANGPTL4 in HCC patients and its therapeutic implication and underlying mechanisms on HCC growth, angiogenesis and metastasis.

## Results

### Underexpression of *ANGPTL4*in HCC

**Figure 1:**
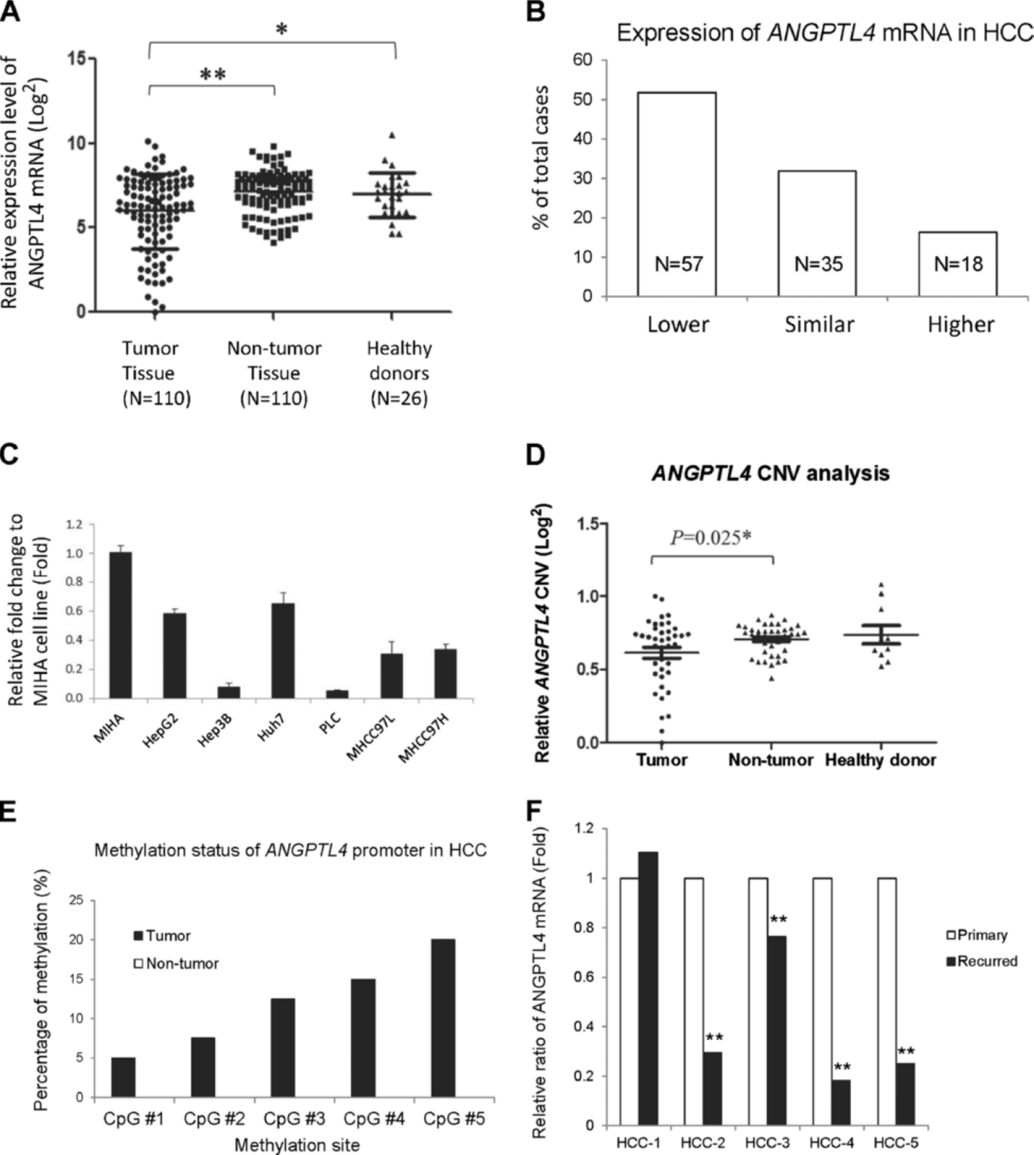
**Downregulation of** ***ANGPTL4*** **gene in HCC. (A)** Expression profile of *ANGPTL4* mRNA among 26 healthy liver tissues and 110 HCC patients. **(B)** Summary of expression difference of *ANGPTL4* mRNA between paired tumor and non-tumor liver tissues among 110 HCC patients. Lower, expression in tumor was 2-fold lower than in non-tumor; Similar, expression in tumor was neither 2-fold higher non 2-fold lower than in non-tumor; Higher, expression in tumor was 2-fold higher than in non-tumor. **(C)** Relative expression level ratio of *ANGPTL4* mRNA in HCC cell lines to normal liver cell line. **(D)** Copy number variation (CNV) analysis of *ANGPTL4* gene in healthy donors and HCC patients. The DNA samples for CNV analysis were randomly selected from those used for mRNA analysis. **(E)** Methylation analysis of *ANGPTL4* promoter in HCC patients by pyrosequencing. **(F)** Relative expression ratio of *ANGPTL4* mRNA between primary and recurred tumor tissues of HCC patients. *, *P* < 0.05; **, *P* < 0.01.

Quantitative RT-PCR was employed to study the expression levels of *ANGPTL4* mRNA in paired tumor and non-tumor liver tissues from 110 HCC patients and in liver tissues from 26 healthy donors. The average expression level (log2 base) of *ANGPTL4* mRNA among tumor, non-tumor and healthy donor liver tissues were 5.98, 7.13 and 6.98 respectively (Figure 1A). The tumor tissues expressed a 2.2-fold lower level of *ANGPTL4* mRNA than non-tumor tissues of HCC patients in average, which was statistically significant using unpaired and paired t-tests (2-tailed unpaired t-test, *p* < 0.0001; 2-tailed paired t-test, *p* < 0.0001). There was no significant difference of *ANGPTL4* mRNA between non-tumor tissues and healthy liver tissues. Comparing paired tumor and non-tumor tissues of each HCC patients, 51.82% (57/110) of tumor tissues were found to have lower expression of *ANGPTL4* mRNA while 16.36% (18/110) of tumor tissues expressed higher *ANGPTL4* mRNA (Figure 1B). The expression levels of *ANGPTL4* mRNA in the 6 HCC cell lines were lower than in MIHA cell line (Figure 1C). These data suggested that the expression of *ANGPTL4* mRNA was commonly downregulated in HCC.

To understand the possible mechanism of downregulation of ANGPTL4 in HCC, copy number variation (CNV) analysis and CpG methylation analysis by pyrosequencing were performed in 40 pairs of tumor and non-tumor DNA samples from HCC patients. The relative *ANGPTL4* CNV value in tumor tissues of HCC patients was determined to be significantly lower than in non-tumor tissues (*p* = 0.025, unpaired two-tailed t-test, Figure 1D). The result from pyrosequencing showed that there were 5 to 20% of positive methylations detected among the 5 CpG sites of the *ANGPTL4* promoter in tumor tissues while there was no positive methylation detected in all non-tumor tissues (Figure 1E). These data suggested that hypermethylation of the promoter region of *ANGPTL4* gene may be one of the mechanisms leading to downregulation of *ANGPTL4* expression in HCC.

### Clinical relevance and prognostic values of *ANGPTL4*in HCC

Statistical analyses were performed to investigate the clinical relevance and prognostic potential of *ANGPTL4* mRNA in HCC. Comparing the expression levels of *ANGPTL4* mRNA between paired primary and recurred tumor tissues in 5 HCC patients, 4 recurred tumors (80%) were found to have significantly lower levels of *ANGPTL4* mRNA than their matched primary tumors (Figure 1F). Statistical analyses showed that either higher degree of downregulation *ANGPTL4* mRNA or lower expression level of *ANGPTL4* mRNA in tumor tissues of HCC patients were significantly associated with presence of venous infiltration, poor differentiation, advanced pathologic tumor-node-metastasis (pTNM) stage and presence of recurrence (Table 1). Moreover, correlation analysis by Pearson coefficient test showed that the expression of *ANGPTL4* mRNA was negatively correlated with serum alpha fetoprotein (AFP) level, and overall and disease-free survivals (Table 2). The above result suggested that the degree of downregulation was positively correlated with advanced stage of HCC and poor survivals of HCC patients. The expression level of *ANGPTL4* mRNA in non-tumor tissues of HCC patients was not significantly associated with any clinical factors (Tables 1 and 2).

**Table 1:** **Statistical analysis of** ***ANGPTL4*** **mRNA with clinical factors of HCC patients using two-tailed T-test**

		***ANGPTL4***mRNA in HCC patients					
		Tumor/non-tumor difference		Tumor		Non-tumor	
Clinical factors	Number (n)	Mean (Log2)	***P***	Mean (Log2)	***p***	Mean (Log2)	***p***
**Sex**							
Male	88	−1.20	0.889	5.85	0.381	7.15	0.670
Female	22	−1.12		6.32		7.02	
**Venous infiltration**							
Absent	51	−0.67	0.023*	6.51	0.012*	7.18	0.692
Present	59	−1.63		5.45		7.08	
**Differentiation** ^**a**^							
Moderately or well	87	−0.82	0.000***	6.21	0.013*	7.17	0.639
Undifferentiated or poorly	18	−3.21		4.80		7.01	
**pTNM staging** ^**a**^							
Early stage	34	−0.35	0.007**	6.67	0.025*	7.04	0.646
Advanced stage	75	−1.56		5.66		7.16	
**Cirrhosis** ^**a**^							
No cirrhosis	39	−0.97	0.479	5.98	0.981	6.98	0.382
Cirrhosis	69	−1.29		5.97		7.20	
**Recurrence**							
No recurrence	49	−0.69	0.035*	6.58	0.006**	7.20	0.577
Recurrence	61	−1.58		5.43		7.07	
**Hepatitis B surface antigen**							
Negative	16	−0.99	0.700	5.53	0.429	6.61	0.080
Positive	94	−1.22		6.01		7.22	

^a^Total number less than 110 due to missing data; *, *p*<0.05; **, *p*<0.01; ***, *p*<0.001.

**Table 2:** **Correlation analysis of ANGPTL4 mRNA with clinical factors of HCC patients**

		***ANGPTL4***mRNA in HCC patients					
		Tumor/non-tumor difference		Tumor		Non-tumor	
Clinical factors	Number (n)	Pearson correlation (R)	***P***	Pearson correlation (R)	***p***	Pearson correlation (R)	***p***
**Age (Year)**	110	−0.039	0.682	−0.084	0.382	−0.038	0.693
**AFP level (mg/ml)**	110	−0.276	0.004**	−0.263	0.005**	0.051	0.593
**Tumor size** ^**a**^ **(cm)**	95	0.017	0.871	0.077	0.456	0.186	0.071
**Overall survival (Month)**	110	0.289	0.002**	0.230	0.016*	−0.123	0.201
**Disease-free survival (Month)** ^**b**^	109	0.241	0.012*	0.266	0.005**	−0.032	0.740

^a^Total number less than 110 due to missing data. ^b^Total number less than 110 due to one patients was out of selection; *, *p*<0.05; **, *p*<0.01.

**Figure 2:**
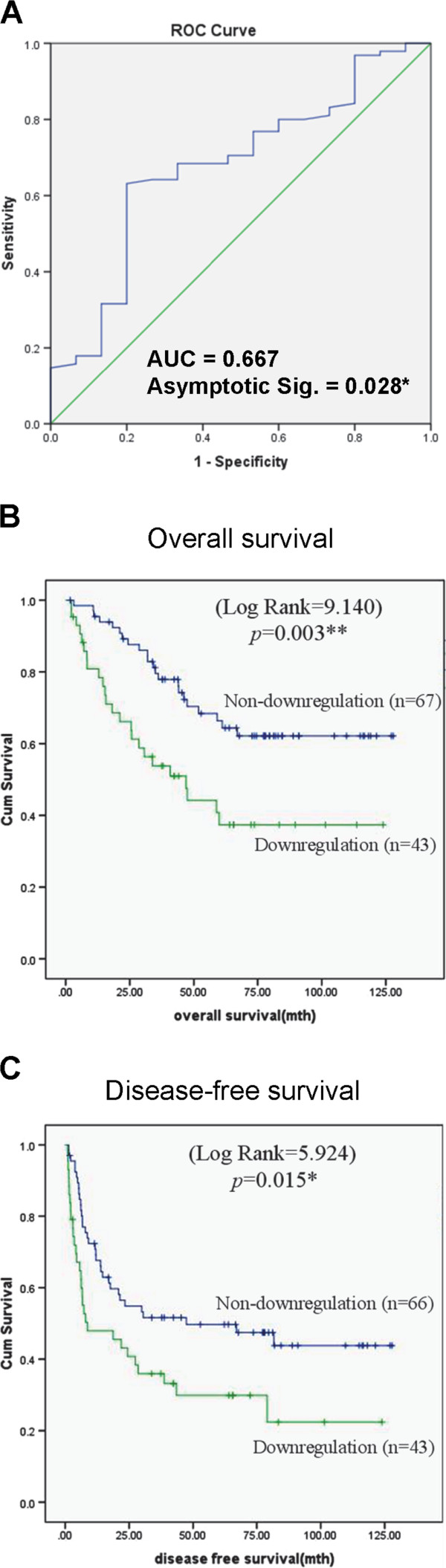
**Survival analyses of** ***ANGPTL4*** **mRNA in HCC patients. (A)** Receiver operating characteristic (ROC) curve analysis of *ANGPTL4* mRNA on predicting 1-year overall survival of HCC patients after liver resection. **(B)** Kaplan-Meier analysis of *ANGPTL4* mRNA on predicting overall survival of HCC patients after hepatectomy. **(C)** Kaplan-Meier analysis of *ANGPTL4* mRNA on predicting disease-free survival of HCC patients after hepatectomy. *, *P* < 0.05; **, *P* < 0.01.

To obtain an optimal cutoff point for examining the prognostic value of *ANGPTL4* mRNA in HCC patients, Receiver operating characteristic (ROC) curve was generated to analyze the sensitivity and 1-specificity using the value of ΔΔΔCt(*ANGPTL4*_patient_) to predict the 1^st^ year postoperative overall survival of HCC patients (Figure 2A). The under area of ROC curve was determined as 0.677. According to Youden index, the optimal cutoff value of ΔΔΔCt(*ANGPTL4*_patient_) among HCC patients was determined to be −1.585. Sixty-seven (60.90%) and 43 (39.09%) HCC patients were defined as *ANGPTL4* mRNA Non-downregulation group and Downregulation group. Kaplan-Meier analysis illustrated that HCC patients in Downregulation group was significantly associated with poor overall survival rate (*p* = 0.003, Figure 2B) and poor disease-free survival rate (*p* = 0.015, Figure 2C) of the patients after hepatectomy compared to patients in Non-downregulation group. The mean periods of postoperative overall and disease-free survivals for Downregulation group were 61.7 and 41.2 months, while for Non-downregulation group were 93.0 and 66.7 months. Univariable Cox regression hazard analysis showed that *ANGPTL4* mRNA was a significant prognostic biomarker for predicting postoperative overall and disease-free survivals of HCC patients (Table 3). The above results suggested that *ANGPTL4* mRNA may be a prognostic biomarker for HCC patients after hepatectomy.

**Table 3:** **Cox proportional hazard regression analysis of** ***ANGPTL4*** **mRNA and clinicopathological parameters in relation to the overall and disease-free survivals of HCC patients after hepatectomy**

	Univariable analysis		Multivariable analysis	
	HR (95% CI)	***P***	HR (95% CI)	***P***
1. Overall survival				
***ANGPTL4*** **mRNA**				
Downregulation *vs* Non-downregulation	2.39(1.33-4.27)	0.003**	1.48(0.81-2.72)	0.204
**pTNM staging**				
Advanced *vs* early	5.37(2.25-12.81)	0.000***	2.26(0.71-7.18)	0.168
**Venous infiltration**				
Presence *vs* absence	4.41(2.25-8.67)	0.000***	2.11(0.88-5.09)	0.096
**AFP level**				
>20 ng/ml *vs* ≤20 ng/ml	3.10(1.60-5.99)	0.001**	2.12(1.07-4.21)	0.031*
2. Disease-free survival				
***ANGPTL4*** **mRNA**				
Downregulation *vs* Non-downregulation	1.83(1.12-2.99)	0.017*	1.40(0.84-2.32)	0.201
**pTNM staging**				
Advanced *vs* early	2.87(1.57-5.24)	0.001**	1.33(0.53-3.35)	0.544
**Venous infiltration**				
Presence *vs* absence	3.14(1.84-5.37)	0.000***	2.36(1.06-5.26)	0.036*

*Abbreviations:* *HR* hazard ratio, *CI* confidence interval; *, *p*<0.05; **, *p*<0.01; ***, *p*<0.001.

### ANGPTL4 suppressed HCC tumorigenesis and metastasis

**Figure 3:**
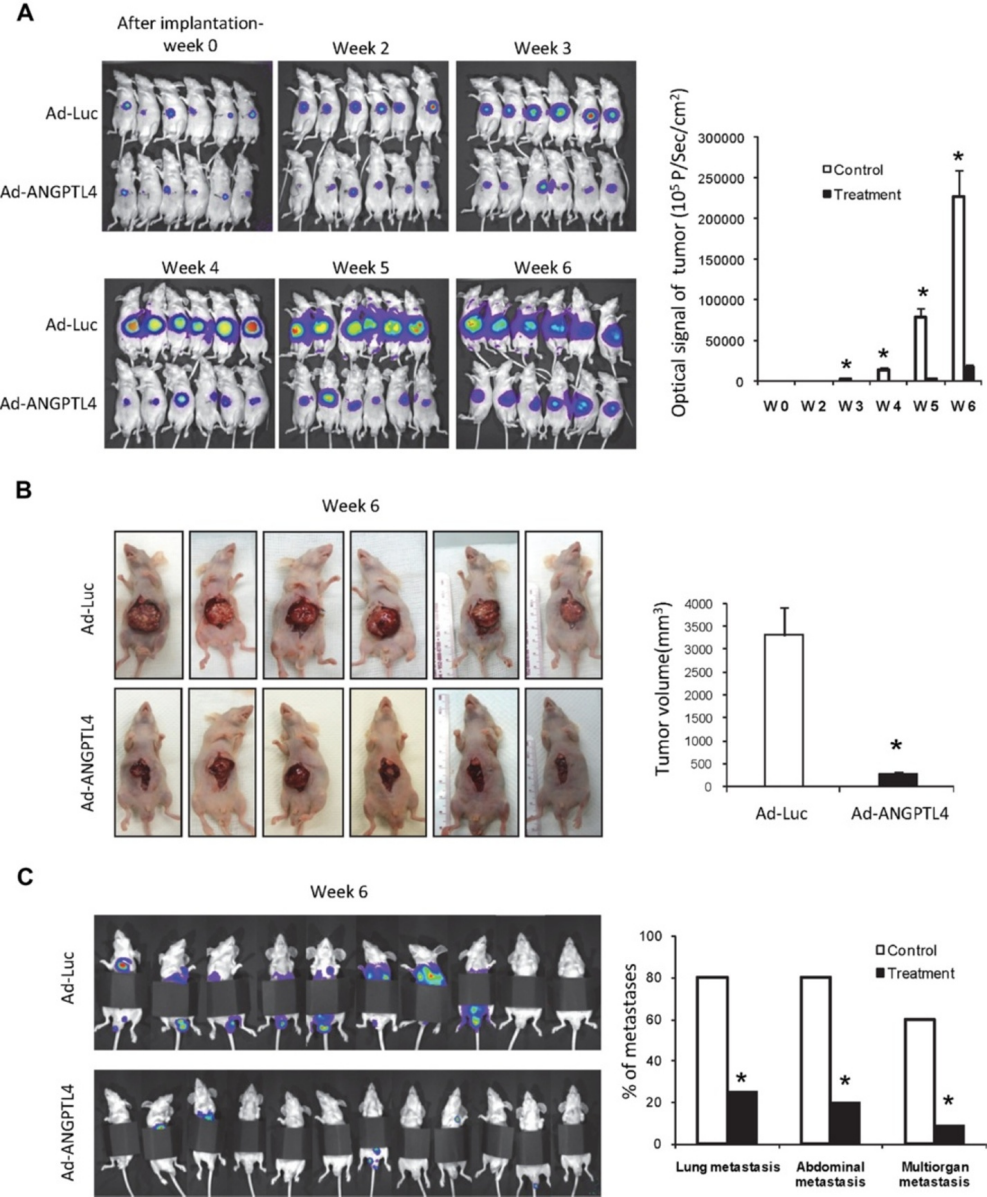
**Therapeutic implication of ANGPTL4 in suppression** ***in vivo*** **tumor growth and metastasis of HCC. (A)** *In vivo* imaging of xenograft liver tumor in nude mice between control and ANGPTL4 treatment groups at week 0, 2, 3, 4, 5 and 6. **(B)** Liver tumor formation at week 6 between control and ANGPTL4 treatment groups **(C)** *In vivo* imaging of metastases on lung at week 6 after covering the luciferase signal from the primary liver tumor. *, *P* < 0.05.

**Figure 4:**
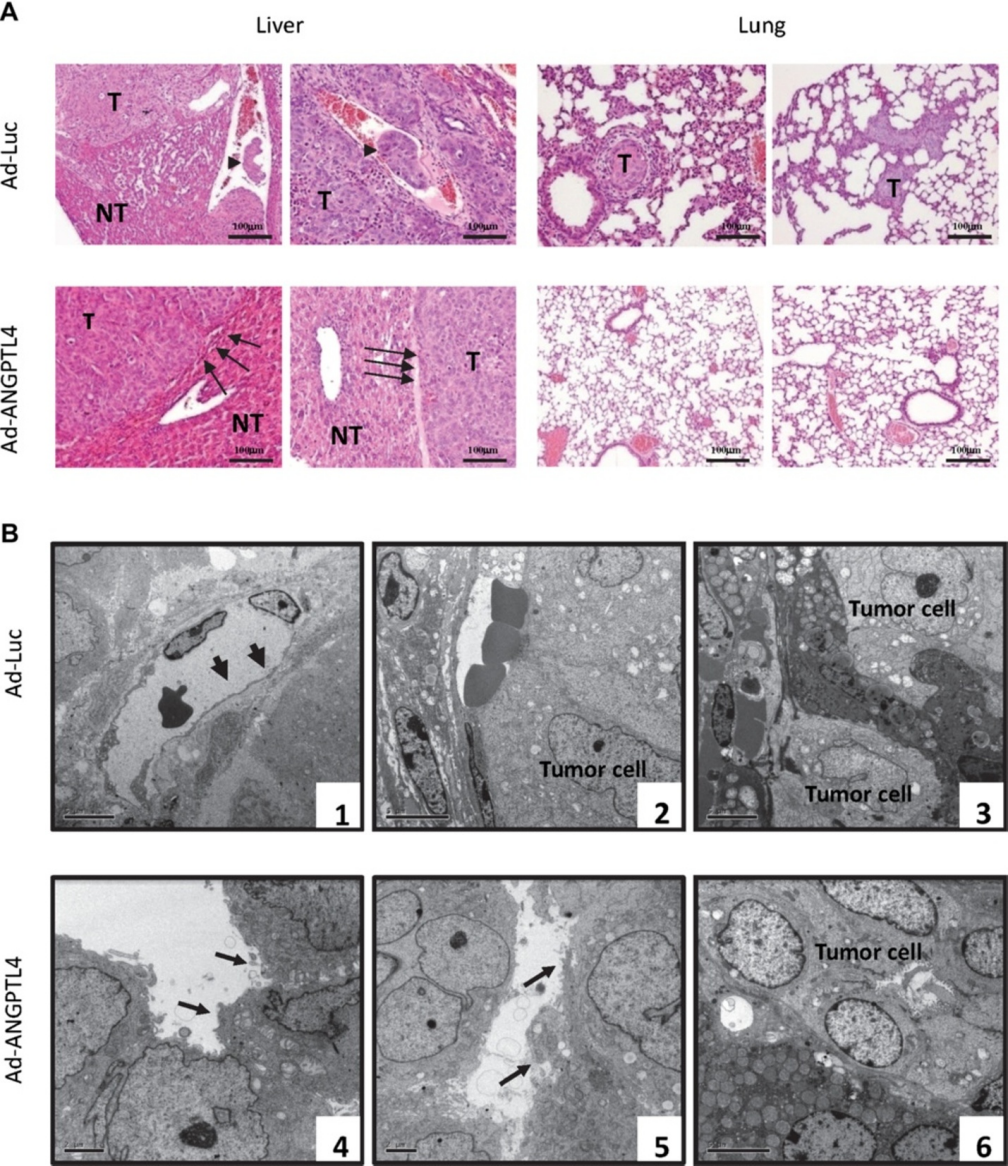
**ANGPTL4 suppressed** ***in vivo*** **invasiveness and metastasis of HCC. (A)** Hematoxylin-eosin staining of liver and lung tissues between control and ANGPTL4 treatment group at week 6. The arrow heads in Ad-Luc group indicate the presence of tumor thrombus in the control group, while the arrowheads in the treatment group indicate the intact encapsulation surrounding the tumor. T, tumor; NT, non-tumor. **(B)** Ultrastructure of liver tumor tissues by transmission electron microscopy. In the control group, the liver tumor was observed to have (1) intact endothelial cells indicated by arrows, (2) infiltrated tumor cells into vessels and (3) invasive phenotype. In Ad-ANGPTL4 treatment group, the liver tumor was observed to have (4 & 5) disrupted endothelial cells indicated by the arrows and (6) intact tumor capsule.

The therapeutic potential of ANGPTL4 treatment was examined in an orthotopic xenograft liver tumor model in mice. The result showed that injection of Ad-ANGPTL4 after orthotopic implantation of human liver tumor significantly hindered tumor formation from 3 weeks to 6 weeks (Figure 3A). The average tumor volume at week 6 was reduced by 11.6 folds after Ad-ANGPTL4 treatment compared to the control group (Figure 3B). Moreover, significant inhibitions of distant metastases including lung, abdominal and multi-organ metastases at week 6 were detected in Ad-ANGPTL4 treatment group compared to the control group (Figure 3C). Histological examination revealed that liver tumors in the control group exhibited progressive morphologies including disrupted encapsulation and infiltrated tumor thrombus, while in Ad-ANGPTL4 treatment group, most of the tumors were surround by intact capsules and without venous-infiltrated tumor thrombus (Figure 4A). Moreover, the non-tumor liver tissues remained normal after Ad-ANGPTL4 treatment (Figure 4A). Histological examination of consecutive lung tissue sections also confirmed a significant reduction of lung metastasis at week 5 after Ad-ANGPTL4 treatment compared to the control group (Figure 4A). Consistently, transmission electron microscopy analysis revealed that Ad-ANGPTL4 treatment disrupted the integrity of the tumor endothelial cells and suppressed the invasiveness of the tumor compared to the control group (Figure 4B). These date suggested that ANGPTL4 may be a potential therapeutic agent to suppress HCC growth and metastasis.

### ANGPTL4 promoted tumor apoptosis and inhibits tumor angiogenesis

**Figure 5:**
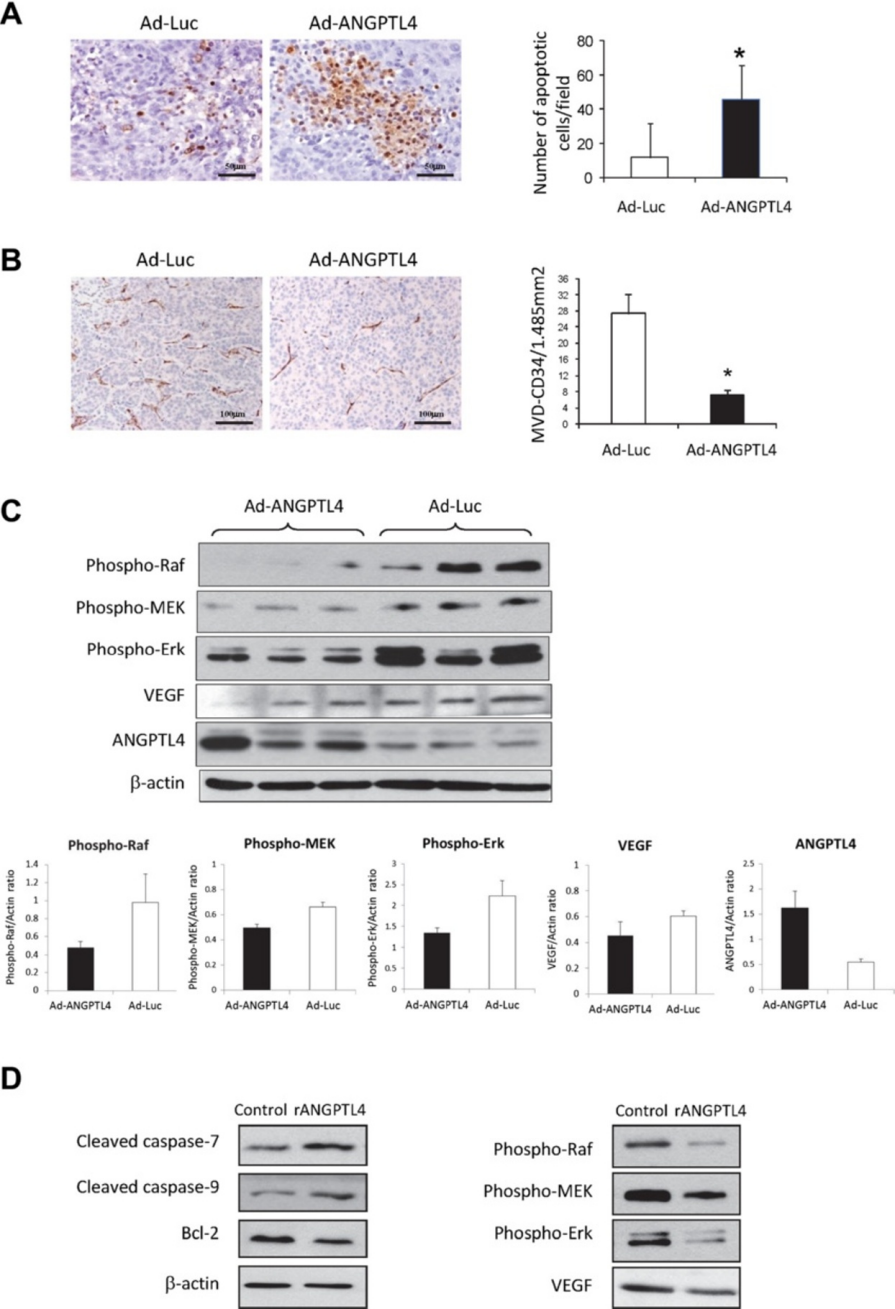
**ANGPTL4 promoted apoptosis and suppressed angiogenesis of HCC. (A)** TUNEL assay on liver tumor tissues. **(B)** Determination of microvessel density (MVD) of liver tumor tissues by immunohistochemical staining using anti-CD34 antibody. **(C)** Western blot analysis of VEGF, phosphor-Raf, Phospho-MEK, Phospho-Erk, ANGPTL4 and beta-actin proteins in liver tumor tissues in Ad-ANGPTL4 and Ad-Luc groups. The average protein expression between Ad-ANGPTL4 and Ad-Luc group was quantified and summarized in the lower panel figures. **(D)** Western blot analysis of proteins in MHCC97L cells after treating with rNGPTL4 protein. *, *P* < 0.05.

To understand the possible mechanisms of ANGPTL4 on suppressing HCC growth, its effects on apoptosis and angiogenesis of HCC were studied. The result showed that Ad-ANGPTL4 treatment significantly increased the number of intratumoral apoptotic cells by approximately 3 folds compared to the control group (Figure 5A). Moreover, administration of recombinant ANGPTL4 (rANGPTL4) protein could increase the activation of caspase 7 and 9, and downregulated the expression of anti-apoptotic protein Bcl2 (Figure 5D). These data suggested that ANGPTL4 could promote apoptosis of HCC cells. In order to study the effect of ANGPTL4 on angiogenesis of HCC, the intratumoral microvessel density (MVD) which indicates the formation of new vessels was determined by immunohistochemical staining of CD34 antibody. The result showed that Ad-ANGPTL4 treatment significantly decrease the MVD by 4 folds in tumor compared to control group (Figure 5B). This suggested ANGPTL4 can suppress angiogenesis of HCC. Furthermore, Western blot analysis showed that Ad-ANGPTL4 treatment could inhibit the expression of VEGF protein and phosphorylation of Raf-MEK-Erk signaling pathway of HCC (Figure 5C). Administration of rANGPTL4 protein on HCC cells also inhibit the expression of VEGF and activation of Raf-MEK-Erk pathways (Figure 5D). These data suggested an anti-angiogenic effect of ANGPTL4 on HCC.

### ANGPTL4 suppressed the motility of HCC cells

**Figure 6:**
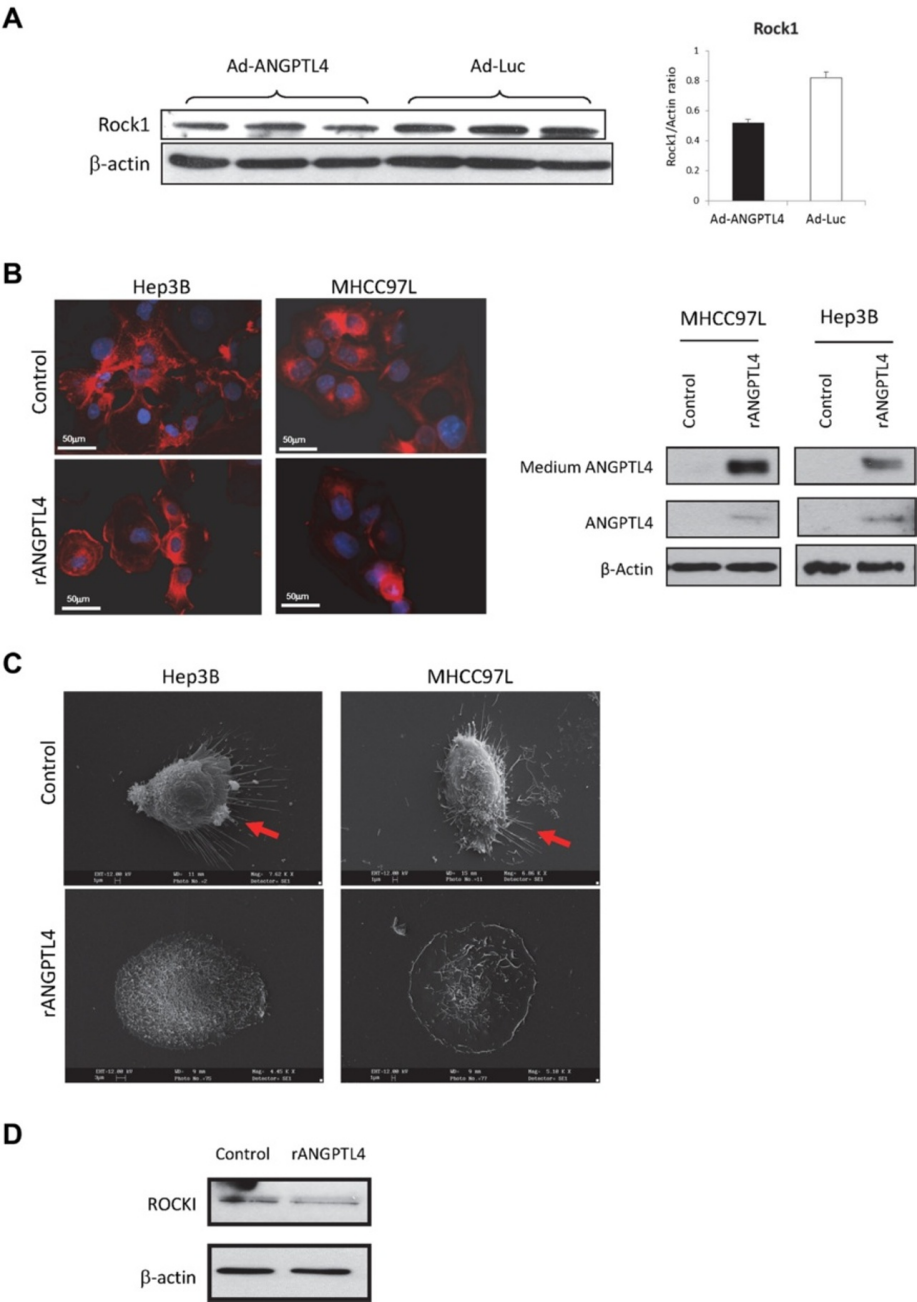
**ANGPTL4 suppressed the motility of HCC. (A)** Western blot analysis of ROCK1 protein in tumor xenograft between Ad-ANGPTL4 treatment group and the Ad-Luc control group. Up-regulation of ANGPTL4 protein by Ad-ANGPTL4 treatment was validated in Figure 5C. **(B)** The effect of rANGPTL4 protein on formation of polymerized stress fibers in HCC cells, indicated by immunostaining of F-actin using rhodamine-phalloidin probe. Red color, F-actin signal; Blue color, nucleic staining by DAPI. The level of ANGPTL4 protein in cells and medium was determined by Western blot analysis **(C)** Scanning electron microscopy of the morphology of HCC cells after administration of rANGPTL4 protein. Red arrows indicate the formation of extracellular “hair-like” stress fibers. **(D)** Western blot analysis of ROCK1 protein in MHCC97L cells after administration of rANGPTL4 protein.

In order to understand the mechanism of ANGPTL4 on suppressing HCC metastasis, its effect on HCC motility was investigated. ROCK1 is an important player in regulating the motility and migration of cancer cells [20]. Western blot analysis showed that treatment of Ad-ANGPTL4 suppressed the expression of ROCK1 protein in tumor tissues compared to the control group (Figure 6A). *In vitro* experiment showed that administration of rANGPTL4 protein on HCC cell lines suppressed the their formation of polymerized stress fibers which indicated by F-actin staining (Figure 6B). Under scanning electronic microscopy, the formation of hair-like fiber structure of Hep3B and MHCC97L cells was suppressed after administration of rANGPTL4 protein (Figure 6C). Western blot analysis showed that rANGPTL4 protein decreased the expression of ROCK1 protein in MHCC97L cells (Figure 6D). These data suggested that ANGPTL4 could suppress the motility of HCC cells thereby reduce their metastatic ability.

### ANGPTL4 influenced tumor-microenvironment

**Figure 7:**
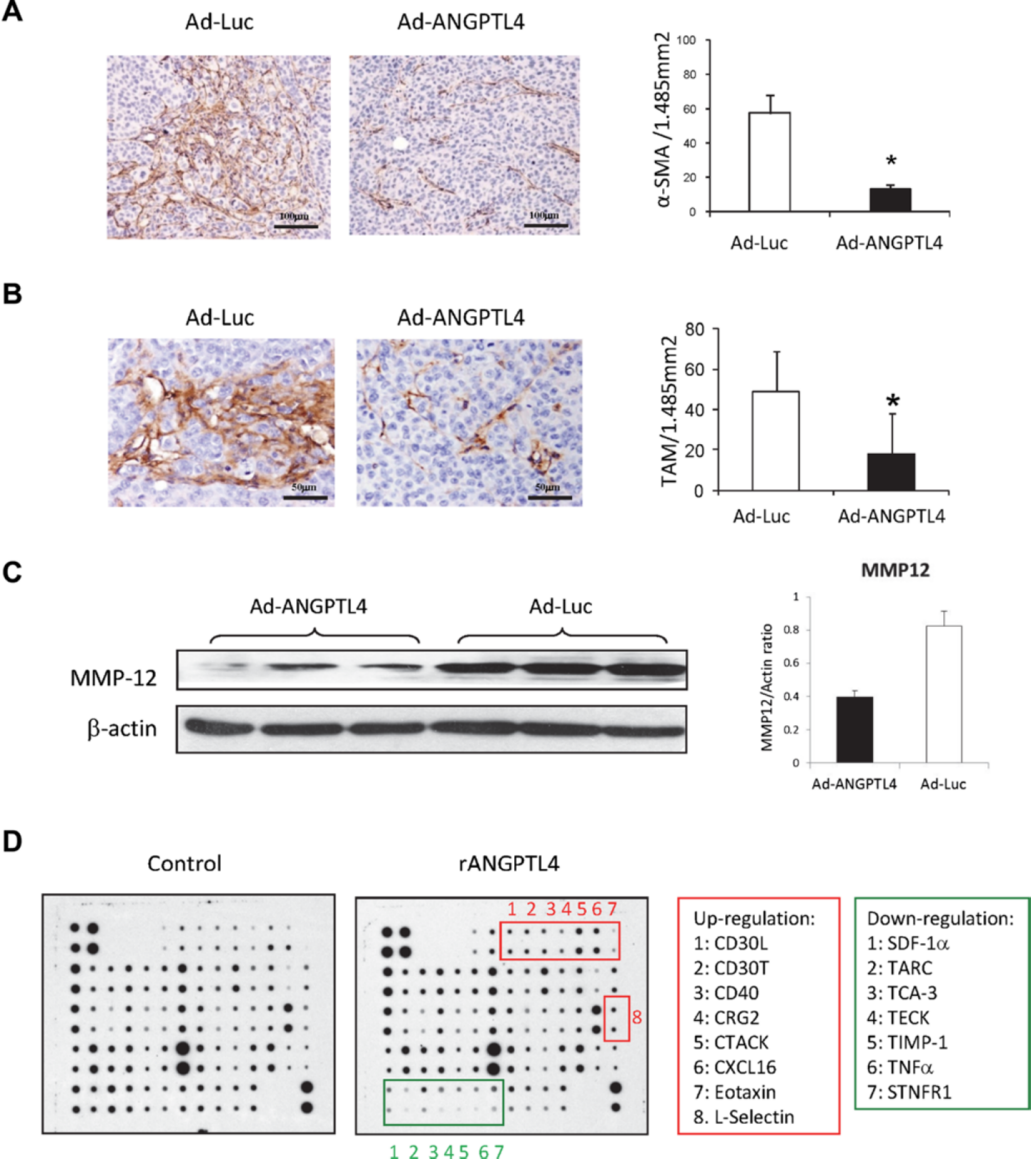
**ANGPTL4 modulated tumor microenvironment. (A)** Immunohistochemical staining of α-SMA protein on liver tumor tissues. **(B)** Determination of tumor associated macrophage (TAM) in liver tumor tissues by immunohistochemical staining of CD68 protein. **(C)** Western blotting of MMP-12 protein in liver tumor tissues between Ad-ANGPTL4 treatment group and Ad-Luc control group. Up-regulation of ANGPTL4 protein by Ad-ANGPTL4 treatment was validated in Figure 5C. **(D)** Cytokine array analysis of the media of Raw264.7 cells. *, *P* < 0.05.

The ability to manipulate the microenvironment is one of the hallmarks of cancer to promote tumor progression, invasion and metastasis [21]. The effect of ANGPTL4 treatment on tumor-microenvironment was therefore studied from different aspects. Compared to the control group, the number of activated hepatic stellate cells (HSCs), revealed by immunohistochemical staining of α-smooth muscle actin (α-SMA) protein, was significantly suppressed after Ad-ANGPTL4 treatment (Figure 7A). The number of infiltrated tumor-associated macrophages (TAMs) stained by anti-CD68 antibody in xenograft liver tumor was significantly reduced after Ad-ANGPTL4 treatment (Figure 7B). Western blot analysis showed that MMP-12 protein was down-regulated in Ad-ANGPTL4 treatment group compared to the control group (Figure 7C). Administration of rANGPTL4 protein to mouse macrophage cell line altered the profile of secreted cytokines including up-regulations of CD30L, CD30T, CD40, CRG2, CTACK, CXCL16, eotaxin and L-selectin, and down-regulations of SDF-1α, TARC, TCA-3, TECK, TIMP-1, TNFα and STNFR1 (Figure 7D). These data suggested that ANGPTL4 can destroy the tumor-favorable microenvironment in HCC.

## Discussion

There have been several studies suggesting that ANGPTL4 is deregulated in cancers, whether it is elevated or suppressed in tumor is dependent on the types and the contexts of cancers [6]. The expression profile of ANGPTL4 in HCC patients is diverse so far. One study has demonstrated that serum ANGPTL4 protein in HCC patients is higher than in chronic hepatitis B patients and normal controls [17]. However the study did not indicate whether the expression of ANGPTL4 in HCC tissues is deregulated or not [17]. Zhu has demonstrated that ANGPTL4 protein is upregulated in tumor tissues of HCC patients compared to normal liver tissue, but only 2 HCC samples were examined on tissue arrays [13]. Another study has found that ANGPTL4 protein in carcinoma tissues is significantly lower than in adjacent tissues of HCC patients [18]. In our study, the overall expression level of *ANGPTL4* mRNA in tumor tissues of HCC patients was lower than non-tumor tissues and healthy liver tissues. Moreover, the number of patients having lower *ANGPTL4* mRNA expression in HCC was greater than the number of having higher *ANGPTL4* mRNA expression in HCC. Furthermore, the relative expression levels of *ANGPTL4* mRNA in 6 HCC cell lines were lower than the normal liver cell line. Therefore, our above evidences suggested that *ANGPTL4* mRNA is underexpressed in HCC. *ANGPTL4* gene locates in chromosome 19p13.3. Several lines of evidences have proved that chromosome 19p is one of the most frequently deleted chromosomal regions in HCC [22–24]. Moreover, a finding in gastric cancer has suggested that methylation is a mechanism of silencing *ANGPTL4* gene leading to tumor development [25]. Methylation of *ANGPTL4* gene has also been found in primary breast cancer [26]. Our CNV and methylation analyses demonstrated that lower copy number of *ANGPTL4* gene and presence of methylation in the promoter of *ANGPTL4* gene might contribute to decreased expression of *ANGPTL4* gene in HCC.

Downregulation of *ANGPTL4* mRNA in HCC was found to be significantly associated with poor prognosis after curative surgery. The clinicopathological parameters of the patients with higher degree of downregulation of *ANGPTL4* were significantly associated with higher malignancy of HCC including advanced HCC stage, presence of venous infiltration, poor differentiation, higher AFP level and appearance of postoperative tumor recurrence. One of the functions of ANGPTL4 is involving in regulating cell differentiation [7]. Our result demonstrated that patients with undifferentiated or poorly differentiated HCC had significantly lower levels of *ANGPTL4* mRNA in tumor tissues (Table 1), suggesting that deregulation of *ANGPTL4* mRNA may indicate the status of tumor differentiation. In addition, most of the recurred HCC tumors expressed lower levels of *ANGPTL4* mRNA than the primary tumors. These above results indicated that the expression level of *ANGPTL4* mRNA in HCC is reversely correlated with tumor malignancy. Most importantly, patients with downregulation of *ANGPTL4* mRNA in HCC were significantly associated with poor postoperative overall and disease-free survivals. Therefore, our data suggested that *ANGPTL4* mRNA may be a potential diagnostic and prognostic biomarker for HCC patients.

ANGPTL4 exhibits both pro-tumorigenic and anti-tumorigenic properties depending on the tissue contexts and the status of posttranslational modifications [6]. Overexpression of ANGPTL4 can inhibit the motility, invasiveness and metastasis of Lewis lung carcinoma and mouse skin cancer cells [16]. However, many studies have suggested that ANGPTL4 can promote tumor growth, angiogenesis, invasion and metastasis [11–15]. A study in HCC has showed that ANGPTL4 promotes tumor migration and metastases [17]. Our study demonstrated that treatment with Ad-ANGPTL4 significantly suppressed not only the *in vivo* growth, angiogenesis and invasiveness, but also the extrahepatic multiorgan metastases of HCC. In addition, treatment with Ad-ANGPTL4 did not cause potential deleterious side effects on the liver tissues. These results suggested that ANGPTL4 may be a potential therapeutic agent for treatment of HCC.

Our study demonstrated several mechanisms of ANGPTL4 in suppressing tumor progression, invasion and metastasis of HCC. First, overexpression of ANGPTL4 suppressed tumor growth through enhancing apoptosis of tumor cells, indicating that suppression of ANGPTL4 in HCC may be a way to escape from apoptosis. Second, overexpression of ANGPTL4 could suppress the invasiveness of HCC cells by restraining its motility through suppression the expression of ROCK1 and formation of polymerized stress fibers. ANGPTL4 is an important regulator involved in vascular permeability and angiogenesis [6, 7]. In this study, Ad-ANGPTL4 treatment significantly suppressed the formation of new vessels in the tumor through repressing the expression of angiogenic factor VEGF and suppressing the activation of Raf-MEK-Erk signaling pathway, suggesting an anti-angiogenic effect of ANGPTL4 on HCC. Angiogenesis which is one of the hallmarks of cancer for obtaining oxygen and nutrients and eliminating wastes is critical for tumorigenesis and metastasis processes [21]. Targeted therapy on inhibiting angiogenesis of HCC has increasingly become one of the important therapeutic strategies in treating patients with advanced HCC [27, 28]. Modulating tumor microenvironment to promote tumor progression, invasion and metastasis is one of the hallmarks of cancer [21]. Myofibroblast which is one of the cancer-associated fibroblasts can be recruited by tumor cells to promote tumor progression and metastasis [21]. Activation of HSCs has been demonstrated to promote HCC progression by generating proinflammatory and proangiogenic microenvironment [29]. In this study, the expression of intratumoral αSMA was suppressed by Ad-ANGPTL4 treatment. Because αSMA is marker for myofibroblast and activated HSCs, suppression of intratumoral αSMA by Ad-ANGPTL4 indicated its potential to inhibit infiltrated myoblasts and the activation of HSCs. HCC is an inflammation-associated cancer in which infiltrated tumor-associated macrophages (TAMs) play critical roles in tumor microenvironment to promote tumorigenesis and progression through secreting cytokines, chemokines and growth factors [30]. MMP-12 which is a proinflammatory factor mainly produced by macrophages is overexpressed in HCC [31]. In our study, the number of infiltrated TAMs was reduced by Ad-ANGPTL4 treatment along with the suppression of MMP-12 expression. Moreover, administration of rANGPTL4 protein could alter the secretions of cytokines from macrophages, indicating its ability to influence the activity of macrophages. Therefore, these results suggested that ANGPTL4 may suppress HCC progression and metastasis through deterioration of tumor-favorable microenvironment. The direct effects and mechanisms of ANGPTL4 on regulating tumor microenvironment of HCC are needed further investigation.

In summary, our data demonstrated that *ANGPTL4* mRNA was commonly underexpressed in HCC. Downregulation of *ANGPTL4* mRNA in HCC was significantly associated with advanced HCC stage, presence of venous infiltration, higher AFP level, poor differentiation, appearance of tumor recurrence, and poor postoperative overall and disease-free survivals of HCC patients. Most importantly, ANGPTL4 treatment could suppress HCC progression and metastasis through promotion of tumor apoptosis, inhibition of tumor motility and angiogenesis, and disruption of tumor-favorable microenvironment. Taken together, our data suggested that ANGPTL4 may be a potential prognostic biomarker and therapeutic agent for patients with advanced HCC.

## Materials and methods

### Patients

Paired tumor and non-tumor liver tissues were recruited from 110 HCC patients undergone liver resection. Paired primary and recurred tumor and non-tumor liver tissues were recruited from 5 HCC patients. Twenty-six normal liver tissues were recruited. The HCC patients were received operation in the Department of Surgery, Queen Marry Hospital, the University of Hong Kong, from December 1999 and October 2007. The study was approved by the Ethics Committee of the University of Hong Kong.

### Cell lines

A human normal liver cell line named MIHA, 4 human HCC cell lines including HepG2, Huh7, PLC and Hep3B, and a mouse macrophage cell line named Raw264.7 were purchased from American Type Culture Collection. Two human metastatic HCC cell lines, MHCC97L and MHCC97H, were the kind gifts from Liver Cancer Institute, Fudan University, Shanghai, China. The cell lines were maintained in Dulbecco’s modified Eagle’s medium (DMEM) with high glucose (Invitrogen) supplemented with 10% heat-inactivated fetal bovine serum (Invitrogen), 100 mg/ml penicillin G and 50 μg/ml streptomycin (Invitrogen) at 37°C in a humidified atmosphere containing 5% CO_2_. MHCC97L cell line stably labeled with the luciferase gene, named MHCC97L-Luc [32], was used for *in vivo* study.

### Orthotopic xenograft nude mice liver tumor model

Male athymic nude mice (BALB/c-nu/nu, 4 – 6 weeks old) were used. Approximately 6 × 10^5^ MHCC97L-Luc cells in 0.2 ml of culture medium were injected subcutaneously in the nude mouse. When the subcutaneous tumor nodule reached 0.8 – 1 cm in diameter, it was removed and cut into cubes about 1 – 2 mm^3^ in size, which were then implanted into the left liver lobes of another group of nude mice, using the method described previously [32, 33]. Then, full-length ANGPTL4-overexpressing adenovirus (Ad-ANGPTL4 treatment group) or Luciferase-overexpressing adenovirus (Ad-Luc control group) was injected into the portal vein of each mouse (10^8^ IU/mouse). The size of the *in vivo* liver tumor and lung metastasis were monitored by Xenogen IVIS® imaging system every week. The mice were sacrificed at 6-weeks after implantation. Six mice were performed for each group. Animal study was approved by Animal (Control of Experiments) Ordinance Chapter 340, the Department of Health, Hong Kong Special Administrative Region (Ref.: (11–371) in DH/HA&P/8/2/3 Pt. 29).

### Real-time quantitative RT-PCR (qRT-PCR)

Total RNA from cells and liver tissues were purified by TriZol Regent (Invitrogen). The quality of RNAs was analyzed by Nanodrop 1000 analyzer (Thermo Scientific) and RNA gel electrophoresis. Method of qRT-PCR analysis was described as in previous study [31]. The expression level of 18S ribosomal RNA and beta-actin was used as the internal control for clinical samples [31, 34] and cells, respectively. Primers used in this study included human *ANGPTL4* gene, sense: 5′-TGACCTCAGATGGAGGCTGGACA-3′, antisense: 5′-CAGCCAGAACTCGCCGTGGG-3′; 18S ribosomal RNA, sense 5′-CTCTTAGCTGAGTGTCCCGC-3′, antisense 5′-CTGATCGTCTTCGAACCTCC-3′; human *beta-actin* gene, sense: 5′-CTCTTCCAGCCTTCCTTCCT-3′, antisense: 5′-AGCACTGTGTTGGCGTACAG-3′. The relative expression level of *ANGPTL4* mRNA for each clinical sample was calculated as: ΔΔCt(*ANGPTL4*_sample_) = ΔCt(*ANGPTL4*_calibrator_) – ΔCt(*ANGPTL4*_sample_), where ΔCt(*ANGPTL4*_calibrator_) = Ct(*ANGPTL4*_calibrator_) – Ct(18S_calibrator_); ΔCt(*ANGPTL4*_sample_) = Ct(*ANGPTL4*_sample_) – Ct(18S_sample_). The calibrator was defined as the sample whose threshold cycle (Ct) value of *ANGPTL4* mRNA was the highest (i.e. sample with the lowest expression level of *ANGPTL4* mRNA) among all samples. The relative expression level of *ANGPTL4* mRNA was presented as relative fold difference in log2 base [31, 35]. The difference of *ANGPTL4* mRNA between tumor and non-tumor tissues of each HCC patient was determined as: ΔΔΔCt(*ANGPTL4*_patient_) = ΔΔCt(*ANGPTL4*_tumor_) – ΔΔCt(*ANGPTL4*_non-tumor_). The value of ΔΔΔCt was equal to 2^-ΔΔΔC^ fold change. PCR analysis for each sample was performed in triplicate.

### Copy number variation (CNV) analysis

Ten DNA samples from healthy donors and forty-pairs of DNA samples from tumor and non-tumor tissues of HCC patients were extracted using Qiagen DNA extraction kit (Qiagen). 10 μg of each DNA sample was used for CNV analysis by using ANGPTL4 specific TaqMan copy number assay (FAM-labeled, Life Technologies). The RNase P gene (VIC-labeled, Life Technologies) was used as control. The PCR analysis was performed by using TaqMan Universal Master Mix II (Life Technologies) and analyzed in the ViiA7 Real Time PCR system (Life Technologies). The relative ANGPTL4 CNV for each sample was determined as ΔΔCt(*ANGPTL4*_sample_) = ΔCt(*ANGPTL4*_calibrator_) – ΔCt(*ANGPTL4*_sample_), where ΔCt(*ANGPTL4*_calibrator_) = Ct(*ANGPTL4*_calibrator_) – Ct(RNaseP_calibrator_); ΔCt(*ANGPTL4*_sample_) = Ct(*ANGPTL4*_sample_) – Ct(RNaseP_sample_). The calibrator is the sample with the lowest ANGPTL CNV. PCR analysis for each samples was performed in triplicate.

### CpG methylation analysis of ANGPTL4 promoter by pyrosequencing

Forty-pairs of DNA samples were extracted from tumor and non-tumor tissues of HCC patients using Qiagen DNA extraction kit (Qiagen). Each DNA was performed bisulfite conversion by EZ DNA Methylation-Direction KIT (Zymo Research). A predesigned ANGPTL4 PyroMark CpG Assay (Hs_ANGPTL4_01_PM, Qiagen) was purchased for quantification of CpG methylation of ANGPTL4 promoter. There were 5 CpG sites in the construct. PCR amplification was performed by using PyroMark® PCR Kit (Qiagen). Pyrosequencing analysis was performed by Genome Research Center, The University of Hong Kong. The sequencing data was analyzed by Pyro Q-CpG Software (Biotage). The percentage of methylation >10% was defined as positive methylation.

### Western blot

Method of Western blot analysis was described in previous study [36]. The amount of 20 μg of total protein from each sample was used for loading. Antibodies including vascular endothelial growth factor (VEGF), ROCK1, Matrix metalloproteinase-12 (MMP-12) and beta-actin antibody were purchased from Santa Cruz Biotechnology (CA). phospho-Raf_(Ser259),_ Phospho-MEK1/2, phosopho-Erk1/2, Bcl-2, cleaved-caspase 7, and cleaved-caspase 9 antibodies were purchased from Cell Signaling Technology. The intensity of Western blot analysis was quantified by Quantity One software (Bio-Rad).

### Morphological study by light and transmission electron microscopy

Liver tumor tissues including non-tumor margin were taken at different time points after tumor implantation for light microscopy with hematoxylin-eosin staining. The specimens were immediately cut into 1-mm cubes and fixed in 2.5% glutaraldehyde in sodium carcodylate hydrochloride buffer overnight at 4°C for electron microscopy section. The sections were then examined under a transmission electron microscope, Philips EM 208 (Koninklijke Philips Electronics N.V., Eindhoven, Netherlands).

### Ultrastructural examination by scanning electron microscopy

Cells treated with or without recombinant ANGPTL4 (rANGPTL4) protein grown on sterile round glass cover slips were fixed with 2.5% glutaraldehyde in 0.1 M sodium cacodylate-HCl buffer, pH 7.4, quenched with 0.1 M sucrose/cacodylate solution, and washed in cacolydate buffer. The samples were then post-fixed with 1% OsO4 in cacodylate buffer. After a cacodylate buffer wash, they were dehydrated through a graded series of ethanol washes, followed by critical point drying using BAL-TEC CPD 030 Critical Point Dryer (BAL-TEC AG, Liechtenstein). The samples were then sputter-coated with a layer of gold using BAL-TEC SCD 005 Sputter Coater (BAL-TEC AG), and visualized using Leica Cambridge Stereoscan 440 SEM (Leica, Cambridge, UK) at an accelerating voltage of 12 kV.

### Immunohistochemistry

Paraffin sections were de-waxed in xylene, rinsed in grade alcohol, and rehydrated in water. Then they were placed in citric buffer (pH 6.0) and treated in a microwave oven with high power for 3 minutes and subsequent low power for 10 minutes. Afterwards, the sections underwent blocking with 3% peroxidase for 20 minutes and 10% goat serum for 30 minutes. Subsequently, primary antibodies with proper dilution were applied on the sections, which were then incubated at 4°C overnight. Following that, secondary antibodies from Dako EnVision™ System (DakoCytomation, Glostrup, Denmark) were applied, and the sections were incubated for 30 minutes at room temperature. Signals were developed with DAB substrate solution (DakoCytomation). The sections were finally counter-stained by hematoxylin solution. Primary antibodies used in this study included VEGF (Santa Cruz Biotechnology), alpha smooth muscle actin (α-SMA, DakoCytomation), CD34 (Santa Cruz Biotechnology), and CD68 (BD Biosciences, San Jose, CA, USA).

### Determination of microvessel density (MVD)

MVD of liver tumor tissue sections was evaluated by immunohistochemical staining with CD34 antibody [37]. Any CD34-positive stained endothelial cell or endothelial cell cluster that was clearly separated from adjacent microvessels, tumor cells and connective elements was counted as one microvessel. The mean microvessel count of the five most vascular areas was taken as the MVD, which was expressed as the absolute number of microvessels per 1.485 mm^2^ (×200 field).

### Terminal deoxynucleotidyl transferase dUTP nick end labeling (TUNEL) assay

Paraffin sections of liver tumor tissues from the treatment group and the control group were detected for apoptotic cells by *In Situ* Cell Death Detection Kit (Roche) according to the manufacturer’s protocol.

### Cytokine array assay

RAW264.7 cells were seeded onto a 6-well plate and incubated at 37°C with 5% CO_2_ for 24 hours. Then the cells were treated with DMEM medium containing 2.5 μg/ml of recombinant mouse ANGPTL4 protein (Obtained from Prof. Aimin Xu, Department of Medicine, the University of Hong Kong) for 24 hours. Cytokine profiling of the medium was analyzed with RayBio® Mouse Cytokine Antibody Array (Cat# AAM-CYT-3, RayBiotech Inc.) according to manufacturer’s instruction.

### Immunofluorescent staining

Cells were fixed with 4% paraformaldehyde in PBS for 15 min at room temperature, and permeabilized with 0.5% Triton X-100 in PBS for 15 min. The cells were blocked with 1% bovine serum albumin in PBS for 30 minutes and then incubated with rhodamine phalloidin probe (Invitrogen) for 1 h at room temperature. After 3 washes in PBS, the cells were stained with DAPI at room temperature for 10 minutes. The cells were washed 3 times with PBS and mounted with FluorSave Reagent (Calbiochem). The slides were analyzed by an image analysis system (Eclipse E600, Nikon).

### Statistical analysis

For clinical samples, the associations of *ANGPTL4* mRNA with clinicopathological parameters were analyzed by two-tailed t-test (Sex, Venous infiltration, differentiation, pTNM staging, cirrhosis, recurrence and hepatitis B surface antigen) or correlation analysis (Age, serum AFP level, tumor size, duration overall survival and duration of disease-free survival). Receiver Operating Characteristic (ROC) curve was generated to analyze the sensitivity and 1-specificity of ΔΔΔCt(*ANGPTL4*_patient_) value to predict 1^st^ year overall survival of HCC patients after hepatectomy. Youden index was used to determine Non-downregulation group and Downregulation group of HCC patients. The prognostic value of *ANGPTL4* mRNA in predicting overall and disease-free survivals of HCC patients after hepatic resection was calculated by Kaplan-Meier analysis with the log-rank test. For disease-free survival analysis, HCC patients under the category of hospital mortality were excluded. Cox proportional hazard regression model was performed with univariable and multivariable analyses to test factors that were significantly associated with the postoperative overall survival and disease-free survival of the HCC patients. For animal study, continuous variables were expressed as median with range. Mann–Whitney *U* test was used for statistical comparison. Chi-square (χ^2^) test was used to compare incidence of lung metastasis in the nude mice orthotopic liver tumor model. Calculations were made with SPSS computer software (SPSS Inc., Chicago, IL, USA). *P* value < 0.05 was considered to be statistically significant.

## Authors’ information

Kevin Tak-Pan Ng, Qualification: Bsc, MPhil, PhD. Current position: Research Assistant Professor.

Aimin Xu, Qualification: PhD. Current position: Professor.

Qiao Cheng, Qualification: MD, PhD.

Dong Yong Guo Qualification: MD, PhD. Current position: Medical Consultant.

Zophia Xue-Hui Lim Qualification: Bsc, PhD. Current position: Scientist.

Chris Kin-Wai Sun Qualification: BSc, PhD. Current position: Research Officer.

Jeffrey Hon-Sing Fung Qualification: BSc. Current position: Research Assistant.

Ronnie Tung-Ping Poon Qualification: MD, PhD. Current position: Chair Professor.

Sheung Tat Fan Qualification: MD, PhD. Current position: Chair Professor.

Chung Mau Lo Qualification: MD. Current position: Chair Professor.

Kwan Man, Qualification: MD, PhD. Current position: Professor.

## Abbreviations

AFP: Alpha fetoprotein
ANGPTL4: Angiopoietin-like 4
CNV: Copy number variation
HCC: Hepatocellular carcinoma
HSC: Hepatic stellate cell
MVD: Microvessel density
pTNM: Pathologic tumor-node-metastasis
qRT-PCR: Quantitative reverse transcriptase polymerase chain reaction
ROC: Receiver operating characteristic
SMA: Smooth muscle actin
TAM: Tumor-associated macrophages
TUNEL: Terminal deoxynucleotidyl transferase dUTP nick end labeling
VEGF: Vascular endothelial growth factor.

## References

[1] Jemal A, Bray F, Center MM, Ferlay J, Ward E, Forman D (2011). Global cancer statistics. CA Cancer J Clin.

[2] Zhu AX (2006). Systemic therapy of advanced hepatocellular carcinoma: how hopeful should we be?. Oncologist.

[3] Worns MA, Weinmann A, Schuchmann M, Galle PR (2009). Systemic therapies in hepatocellular carcinoma. Dig Dis.

[4] Yau T, Chan P, Epstein R, Poon RT (2008). Evolution of systemic therapy of advanced hepatocellular carcinoma. World J Gastroenterol.

[5] Yau T, Chan P, Epstein R, Poon RT (2009). Management of advanced hepatocellular carcinoma in the era of targeted therapy. Liver Int.

[6] Tan MJ, Teo Z, Sng MK, Zhu P, Tan NS (2012). Emerging roles of Angiopoietin-like 4 in human cancer. Mol Cancer Res.

[7] Zhu P, Goh YY, Chin HF, Kersten S, Tan NS (2012). Angiopoietin-like 4: a decade of research. Biosci Rep.

[8] Kersten S, Lichtenstein L, Steenbergen E, Mudde K, Hendriks HF, Hesselink MK, Schrauwen P, Muller M (2009). Caloric restriction and exercise increase plasma ANGPTL4 levels in humans via elevated free fatty acids. Arterioscler Thromb Vasc Biol.

[9] Mandard S, Zandbergen F, van Straten E, Wahli W, Kuipers F, Muller M, Kersten S (2006). The fasting-induced adipose factor/angiopoietin-like protein 4 is physically associated with lipoproteins and governs plasma lipid levels and adiposity. J Biol Chem.

[10] Lu B, Moser A, Shigenaga JK, Grunfeld C, Feingold KR (2010). The acute phase response stimulates the expression of angiopoietin like protein 4. Biochem Biophys Res Commun.

[11] Nakayama T, Hirakawa H, Shibata K, Nazneen A, Abe K, Nagayasu T, Taguchi T (2011). Expression of angiopoietin-like 4 (ANGPTL4) in human colorectal cancer: ANGPTL4 promotes venous invasion and distant metastasis. Oncol Rep.

[12] Zhang H, Wong CC, Wei H, Gilkes DM, Korangath P, Chaturvedi P, Schito L, Chen J, Krishnamachary B, Winnard PT, Raman V, Zhen L, Mitzner WA, Sukumar S, Semenza GL (2012). HIF-1-dependent expression of angiopoietin-like 4 and L1CAM mediates vascular metastasis of hypoxic breast cancer cells to the lungs. Oncogene.

[13] Zhu P, Tan MJ, Huang RL, Tan CK, Chong HC, Pal M, Lam CR, Boukamp P, Pan JY, Tan SH, Kersten S, Li HY, Ding JL, Tan NS (2011). Angiopoietin-like 4 protein elevates the prosurvival intracellular O2(−):H2O2 ratio and confers anoikis resistance to tumors. Cancer Cell.

[14] Ma T, Jham BC, Hu J, Friedman ER, Basile JR, Molinolo A, Sodhi A, Montaner S (2010). Viral G protein-coupled receptor up-regulates Angiopoietin-like 4 promoting angiogenesis and vascular permeability in Kaposi’s sarcoma. Proc Natl Acad Sci U S A.

[15] Kim SH, Park YY, Kim SW, Lee JS, Wang D, DuBois RN (2011). ANGPTL4 induction by prostaglandin E2 under hypoxic conditions promotes colorectal cancer progression. Cancer Res.

[16] Galaup A, Cazes A, Le Jan S, Philippe J, Connault E, Le Coz E, Mekid H, Mir LM, Opolon P, Corvol P, Monnot C, Germain S (2006). Angiopoietin-like 4 prevents metastasis through inhibition of vascular permeability and tumor cell motility and invasiveness. Proc Natl Acad Sci U S A.

[17] Li H, Ge C, Zhao F, Yan M, Hu C, Jia D, Tian H, Zhu M, Chen T, Jiang G, Xie H, Cui Y, Gu J, Tu H, He X, Yao M, Liu Y, Li J (2011). Hypoxia-inducible factor 1 alpha-activated angiopoietin-like protein 4 contributes to tumor metastasis via vascular cell adhesion molecule-1/integrin beta1 signaling in human hepatocellular carcinoma. Hepatology.

[18] Zhang H, Wei S, Ning S, Jie Y, Ru Y, Gu Y (2013). Evaluation of TGFbeta, XPO4, elF5A2 and ANGPTL4 as biomarkers in HCC. Exp Ther Med.

[19] Xu A, Lam MC, Chan KW, Wang Y, Zhang J, Hoo RL, Xu JY, Chen B, Chow WS, Tso AW, Lam KS (2005). Angiopoietin-like protein 4 decreases blood glucose and improves glucose tolerance but induces hyperlipidemia and hepatic steatosis in mice. Proc Natl Acad Sci U S A.

[20] Narumiya S, Tanji M, Ishizaki T (2009). Rho signaling, ROCK and mDia1, in transformation, metastasis and invasion. Cancer Metastasis Rev.

[21] Hanahan D, Weinberg RA (2011). Hallmarks of cancer: the next generation. Cell.

[22] Guan XY, Fang Y, Sham JS, Kwong DL, Zhang Y, Liang Q, Li H, Zhou H, Trent JM (2000). Recurrent chromosome alterations in hepatocellular carcinoma detected by comparative genomic hybridization. Gene Chromosome Canc.

[23] Okabe H, Ikai I, Matsuo K, Satoh S, Momoi H, Kamikawa T, Katsura N, Nishitai R, Takeyama O, Fukumoto M, Yamaoka Y (2000). Comprehensive allelotype study of hepatocellular carcinoma: potential differences in pathways to hepatocellular carcinoma between hepatitis B virus-positive and -negative tumors. Hepatology.

[24] Qin LX, Tang ZY, Ye SL, Liu YK, Ma ZC, Zhou XD, Wu ZQ, Lin ZY, Sun FX, Tian J, Guan XY, Pack SD, Zhuang ZP (2001). Chromosome 8p deletion is associated with metastasis of human hepatocellular carcinoma when high and low metastatic models are compared. J Cancer Res Clin Oncol.

[25] Kaneda A, Kaminishi M, Yanagihara K, Sugimura T, Ushijima T (2002). Identification of silencing of nine genes in human gastric cancers. Cancer Res.

[26] Hattori N, Okochi-Takada E, Kikuyama M, Wakabayashi M, Yamashita S, Ushijima T (2011). Methylation silencing of angiopoietin-like 4 in rat and human mammary carcinomas. Cancer Sci.

[27] Zhu AX, Duda DG, Sahani DV, Jain RK (2011). HCC and angiogenesis: possible targets and future directions. Nat Rev Clin Oncol.

[28] Wysocki PJ (2010). Targeted therapy of hepatocellular cancer. Expert Opin Investig Drugs.

[29] Coulouarn C, Corlu A, Glaise D, Guenon I, Thorgeirsson SS, Clement B (2012). Hepatocyte-stellate cell cross-talk in the liver engenders a permissive inflammatory microenvironment that drives progression in hepatocellular carcinoma. Cancer Res.

[30] Capece D, Fischietti M, Verzella D, Gaggiano A, Cicciarelli G, Tessitore A, Zazzeroni F, Alesse E (2013). The inflammatory microenvironment in hepatocellular carcinoma: a pivotal role for tumor-associated macrophages. Biomed Res Int.

[31] Ng KT, Qi X, Kong KL, Cheung BY, Lo CM, Poon RT, Fan ST, Man K (2011). Overexpression of matrix metalloproteinase-12 (MMP-12) correlates with poor prognosis of hepatocellular carcinoma. Eur J Cancer.

[32] Sun CK, Man K, Ng KT, Ho JW, Lim ZX, Cheng Q, Lo CM, Poon RT, Fan ST (2008). Proline-rich tyrosine kinase 2 (Pyk2) promotes proliferation and invasiveness of hepatocellular carcinoma cells through c-Src/ERK activation. Carcinogenesis.

[33] Man K, Ng KT, Xu A, Cheng Q, Lo CM, Xiao JW, Sun BS, Lim ZX, Cheung JS, Wu EX, Sun CK, Poon RT, Fan ST (2010). Suppression of liver tumor growth and metastasis by Adiponectin in nude mice through inhibition of tumor angiogenesis and downregulation of Rho Kinase/IFN-inducible protein 10/Matrix metalloproteinase 9 signaling. Clin Cancer Res.

[34] Ng KT, Man K, Sun CK, Lee TK, Poon RT, Lo CM, Fan ST (2006). Clinicopathological significance of homeoprotein Six1 in hepatocellular carcinoma. Br J Cancer.

[35] Ng KT, Lo CM, Guo DY, Qi X, Li CX, Geng W, Liu XB, Ling CC, Ma YY, Yeung WH, Shao Y, Poon RT, Fan ST, Man K (2014). Identification of transmembrane protein 98 as a novel chemoresistance-conferring gene in hepatocellular carcinoma. Mol Cancer Ther.

[36] Ng KT, Guo DY, Cheng Q, Geng W, Ling CC, Li CX, Liu XB, Ma YY, Lo CM, Poon RT, Fan ST, Man K (2012). A garlic derivative, S-allylcysteine (SAC), suppresses proliferation and metastasis of hepatocellular carcinoma. PLoS ONE.

[37] Poon RT, Ng IO, Lau C, Yu WC, Yang ZF, Fan ST, Wong J (2002). Tumor microvessel density as a predictor of recurrence after resection of hepatocellular carcinoma: a prospective study. J Clin Oncol.

